# Effect of linagliptin plus insulin in comparison to insulin alone on metabolic control and prognosis in hospitalized patients with SARS-CoV-2 infection

**DOI:** 10.1038/s41598-021-04511-1

**Published:** 2022-01-11

**Authors:** Rodolfo Guardado-Mendoza, Miguel Angel Garcia-Magaña, Liz Jovanna Martínez-Navarro, Hilda Elizabeth Macías-Cervantes, Rodolfo Aguilar-Guerrero, Erick L. Suárez-Pérez, Alberto Aguilar-García

**Affiliations:** 1 Research Department, Hospital Regional de Alta Especialidad del Bajío, and University of Guanajuato, Blvd.Milenio #130, Col. San Carlos la Roncha, CP 37660 León, Guanajuato Mexico; 2 Internal Medicine Department, Hospital Regional de Alta Especialidad del Bajío, León, Guanajuato Mexico; 3 Internal Medicine Department, Unidad Médica de Alta Especialidad T1, Instituto Mexicano del Seguro Social, León, Guanajuato Mexico; 4 Department of Biostatistics and Epidemiology, Graduated School of Public Health, University of Puerto Rico, San Juan, USA; 5 Endocrinology Department, Hospital Regional de Alta Especialidad del Bajío, León, Guanajuato Mexico

**Keywords:** Type 2 diabetes, SARS-CoV-2

## Abstract

To evaluate the effect of the combination of linagliptin and insulin on metabolic control and prognosis in hospitalized patients with severe acute respiratory syndrome coronavirus 2 (SARS-CoV-2) infection and hyperglycemia. A parallel double-blind randomized clinical trial including hospitalized patients with SARS-CoV-2 infection and hyperglycemia, randomized to receive 5 mg linagliptin + insulin (*LI group*) or insulin alone (*I group*) was performed. The main outcomes were the need for assisted mechanical ventilation and glucose levels during hospitalization. Subjects were screened for eligibility at hospital admission if they were not with assisted mechanical ventilation and presented hyperglycemia, and a total of 73 patients with SARS-CoV-2 infection and hyperglycemia were randomized to the *LI group* (n = 35) or *I group* (n = 38). The average hospital stay was 12 ± 1 vs 10 ± 1 days for the *I* and *LI groups*, respectively (p = 0.343). There were no baseline clinical differences between the study groups, but the percentage of males was higher in the *LI group* (26 vs 18, p = 0.030). The improvements in fasting and postprandial glucose levels were better in the *LI group* that the I group (122 ± 7 vs 149 ± 10, p = 0.033; and 137 ± 7 vs 173 ± 12, p = 0.017, respectively), and insulin requirements tended to be lower in the *LI group* than the I group. Three patients in the *LI group* and 12 in the *I group* required assisted mechanical ventilation (HR 0.258, CI 95% 0.092–0.719, p = 0.009); 2 patients in the *LI group* and 6 in the *I group* died after a follow-up of 30 days (p = 0.139). No major side effects were observed. The combination of linagliptin and insulin in hospitalized patients with SARS-CoV-2 infection and hyperglycemia reduced the relative risk of assisted mechanical ventilation by 74% and improved better pre and postprandial glucose levels with lower insulin requirements, and no higher risk of hypoglycemia.

This study is registered at clinicaltrials.gov, number NCT04542213 on 09/03/2020.

## Introduction

Coronavirus disease 2019 (COVID-19) is caused by severe acute respiratory syndrome coronavirus 2 (SARS-CoV-2) infection and has reached pandemic status, challenging health systems around the world. SARS-CoV-2 infection is a highly transmissible viral infection caused by a coronavirus that uses angiotensin converting enzyme 2 (ACE2) on the surface of host cells as a receptor and is widely distributed in the respiratory tract and intestinal tract^1^. The main clinical symptoms of COVID-19 are fever, cough and general malaise, among others, and up to more than 50% of patients may be asymptomatic. Other possible symptoms are nausea, loss of appetite, diarrhea, and hyposmia. A diagnosis is usually made according to history of exposure, clinical symptoms, and confirmation through virus detection by RT-PCR. It is important to note that to date there is no definitive and effective treatment for SARS-CoV-2 infection^2^.

T2D is a chronic disease characterized by insulin resistance, pancreatic beta cell dysfunction, pancreatic alpha cell dysfunction, amyloid deposits, a reduction in the incretin effect, etc., which together contribute to the development of hyperglycemia^3^. T2D is associated with a high risk of different types of infections^4^. In addition to the fact that patients with type 2 diabetes have greater susceptibility to all kinds of infection as well as a greater risk of complications once they present acute decompensation, there is evidence that type 2 diabetes is a poor prognostic factor in patients with SARS-CoV-2 infection^5^. Approximately 15–35% of patients hospitalized for SARS-CoV-2 infection also present T2D, and it is likely that a greater percentage of these patients present a high risk of hyperglycemia or early disturbances in glucose metabolism that have not yet been identified.

Recent reports have found that type 2 diabetes, obesity and age are the main variables associated with a worse prognosis in patients with SARS-CoV-2 infection^6–10^. Additionally, patients with type 2 diabetes and COVID-19 show the worst prognosis if they have higher C-reactive protein (CRP) levels and if they were using insulin previously^11^. SARS-CoV-2 has been shown to induce inflammation, insulin resistance and endothelial dysfunction through different mechanisms in patients with hyperglycemia and type 2 diabetes^12,13^.

Hyperglycemia in hospitalized patients results in greater morbidity and mortality^14^, and this has also been observed in patients with T2D and COVID-19^15^. The management of hospitalized patients with hyperglycemia can be complicated by various circumstances, and they are usually treated with insulin. In Mexico, a high number of SARS-CoV-2 infections have been reported, and a large percentage of these patients have T2D, leading to acute decompensation with hyperglycemia, hospitalization, and a high risk of fatal complications.

In recent years, the usefulness of new drugs in hospitalized patients with hyperglycemia has been further evaluated^16^. Dipeptidyl peptidase type-4 (DPP-4) is an enzyme found on the cell surface that interacts with different peptide hormones in the regulation of the immune response^17,18^. One of its main known effects is the inactivation of endogenous incretins (GLP-1 and GIP), which increases the half-life of endogenous GLP-1, stimulates insulin secretion by pancreatic beta cells and reduces glucagon secretion by pancreatic alpha cells^19^. Different studies have also shown the usefulness of DPP-4 inhibitors (linagliptin) both in preventing T2D in patients with prediabetes^20^, and in reducing insulin requirements and improving metabolic control in patients with in-hospital hyperglycemia after kidney transplantation when combined with insulin^16^, and they are mainly indicated for uncontrolled patients with T2D who do not meet glucose control^21,22^.

DPP-4 has been associated with inflammation and its soluble levels have been reported to be both reduced and elevated in different inflammatory processes^23–25^. DPP-4 inhibitors have no major side effects are generally well tolerated and have a good metabolic effect in patients with T2D^26–30^.

DPP-4 has also been documented to function as a receptor for coronavirus^31^, and some studies in animal models have shown that through DPP-4 inhibition, MERS-CoV infection can be alleviated. Additionally, MERS-CoV infection is lethal in transgenic DPP-4 mice^32–35^. Recent studies in in vitro models suggest that DPP-4 is a SARS-CoV coreceptor^36^, and higher expression of DPP-4, even higher than that of ACE2, has been found in different tissues^37^.

Experimental studies have documented that the use of some DPP-4 inhibitors reduces the inflammatory response in different clinical settings^38,39^. DPP-4 inhibitors improve metabolic control and postprandial blood glucose peaks and have a low risk of causing hypoglycemia and good tolerability, making these drugs attractive for use in hospitalized patients. Furthermore, different studies have shown an anti-inflammatory effect of DPP-4 inhibition in different models of T2D^40,41^, although in a case–control study it was not documented that exposure to DPP-4 inhibitors had any role in preventing or reducing the risk of SARS-CoV-2 infection^42^.

Recent retrospective studies have reported an association between the use of sitagliptin, a DPP-4 inhibitor, and reduced mortality and other outcomes in patients with T2D hospitalized for COVID-19^43,44^.

Considering all this together, there is a scientific questions about the beneficial effect that the use of DPP-4 inhibitors could have in patients with COVID-19, however, no prospective and comparative studies have been performed to more clearly dilucidated this scientific concern.

The goal of this work was to evaluate the effect of linagliptin + insulin compared to that of insulin alone on glycemic control and prognosis in hospitalized patients with SARS-CoV-2 infection and hyperglycemia, hypothesizing that the use of DPP-4 inhibitor would improve metabolic control and clinical prognosis.

## Materials and methods

### Study design and participants

This was a parallel randomized clinical trial performed in two third level hospitals of the Center of Mexico that included hospitalized patients with SARS-CoV-2 infection and hyperglycemia with or without previous diagnosis of T2D. Eighty six patients were screened at the time of hospitalization at the Internal Medicine Service of the Hospital Regional de Alta Especialidad del Bajío and at the Unidad Médica de Alta Especialidad T1 from the Instituto Mexicano del Seguro Social in León, Guanajuato, México between June 2020 and February 2021. Patients were eligible for the study if they met the following criteria: (i) patients hospitalized with SARS-CoV-2 infection confirmed by RT-PCR who required supplementary oxygen, (ii) patients with or without prior T2D and plasma glucose levels between 140 and 400 mg/dl, and (iii) patients of both sexes who were older than 18 years of age and were able to take pills orally. Patients were excluded if they were pregnant or if they were already on assisted mechanical ventilation. Of the 86 patients screened, 74 met the selection criteria and were randomly assigned to receive the basal-bolus insulin scheme or to the basal-bolus insulin schema plus linagliptin.

Written informed consent was obtained from all participants and the study was approved by the Research and Ethical Committee at the Hospital Regional de Alta Especialidad del Bajío (CEI-22-2020 and CI-HRAEB-42-2020) and registered at Clinicaltrials.gov NCT04542213 on 09/03/2021. All methods were performed in accordance with the Research Guidelines by the National Health System, as well as in accordance with the International Research Guidelines and the Good Clinical Practice Standards.

### Procedures

#### SARS-CoV-2 diagnosis and biochemical measurements

SARS-CoV-2 infection was diagnosed based on clinical symptoms and confirmation by RT-PCR. Fasting glucose levels were measured by dry chemistry with the colorimetric method (Vitros 5600; Ortho Clinical Diagnostics), and pre- and postprandial glucose levels were measured by the capillary method using an Accu-Chek glucometer. Hypoglycemia was defined as a glucose level ˂70 mg/dl (3.88 mmol/l). Glycated hemoglobin A_1C_ (HbA_1C_) values were determined using high-performance liquid chromatography with a DS-5 Analyzer (Drew Scientific, Inc., Miami, FL, USA). Lipid levels were measured by dry chemistry with the colorimetric method (Vitros 5600, Ortho Clinical Diagnostics), C reactive protein (CRP) levels were measured by a chemiluminescent immunometric assay (Vitros 350), D dimer levels were measured by fluorescence immunoassay (Quidel Cardiovascular Inc., CA USA) and fibrinogen levels were measured by the Clauss method (Instrumentation Laboratory Company, Bedford MA, USA).

##### Randomization and masking

Randomization was carried out by blocks using an electronic random numbers system, and it was performed by a physician not involved in the study. The physicians providing patient care, researchers and personnel who collected and analyzed the data and outcome variables were blinded to the treatment group assignments. We did not use a placebo pill.

##### Interventions

All patients received standard therapy plus the recommended treatment for hospitalized patients with COVID-19^45^. Patients assigned to the linagliptin + insulin group (*LI group*) received 5 mg linagliptin daily plus a basal bolus insulin scheme; patients assigned to the insulin group (*I group*) received only a basal bolus insulin scheme. All treatments were obtained from the respective hospitals. The basal bolus insulin regimen was started and adjusted according to international guidelines; in general, patients received a starting insulin dose of approximately 0.5 U/kg/day, given half as basal insulin (NPH or glargine) once or twice daily and half as insulin lispro divided into three equal doses before meals. The insulin dose was adjusted daily to achieve the goal of a fasting glucose level between 80 and 140 mg/dl (4.44–7.77 mmol/l) or a random glucose level below 180 mg/dl (9.99 mmol/l). The insulin dose was corrected before each meal depending on glucose measurements, increasing 1 unit for each 40 mg above 140 mg/dl (7.77 mmol/l) of glucose. Glucose levels were monitored at fasting and before each meal, as well as at bedtime according to standard clinical practice. Data regarding pre- and postprandial glucose levels, hypoglycemia, clinical evolution and received therapies were recorded from the start of the study until patient discharge.

##### Follow-up and outcomes

Patients were followed during hospitalization for up to 30 days. General treatment for COVID-19 consisted of supplementary oxygen, 6 mg IV dexamethasone every 24 h, prophylactic anticoagulation therapy, and antipyretic therapy, as recommended by international guidelines^45^. Assisted mechanical ventilation was indicated by the physicians in charge of patient care if the patient presented respiratory failure with a respiratory frequency ˃30 per minute, oxygen saturation < 90% despite support with supplementary oxygen, hemodynamic instability, or neurologic deterioration. There were two primary outcomes: (1) need for assisted mechanical ventilation and, (2) mortality. Secondary outcomes were glucose levels and insulin requirements during the first 5–10 days in the hospital, pulmonary parameters and clinical evolution from COVID-19 based on a seven-category ordinal scale consisted of the following categories: 1, not hospitalized and normal activities; 2, not hospitalized, but unable to return to normal activities; 3, hospitalized, not requiring supplemental oxygen; 4, hospitalized, requiring supplemental oxygen; 5, hospitalized, requiring nasal high-flow oxygen therapy, noninvasive mechanical ventilation, or both; 6, hospitalized, requiring invasive mechanical ventilation, or both; and 7, death^46^; hypoglycemia, and systemic inflammation.

### Statistical analysis

#### Sample size and statistical analysis

The sample size was calculated to achieve a minimum difference of 28% between the study groups (41 vs 13%) in mortality and need of mechanical ventilation. Based on previous observational and retrospective reports by Solerte and Mirani, on the effect of DPP-4 inhibitors on prognosis in hospitalized patients with COVID-19 a minimum of 35 patients per group were needed for 5% significance level and 80% statistical power and considering a 20% of expected loss during follow-up^43,44^. This sample size had also the statistical power to find any significant difference in glucose levels between groups, considering data from a previous study carried out in hospitalized kidney transplant patients^16^, in which it was observed that the linagliptin + insulin group had a final glucose level of 135 ± 14 mg/dl whereas the insulin-only group had a glucose level of 155 ± 19 mg/dl.

Normal distribution was confirmed for all the quantitative variables by the Kolmogorov–Smirnov and Shapiro–Wilk tests. Intergroup comparisons at different time points were analyzed by t test for independent groups, and intragroup comparisons were analyzed by repeated measures ANOVA. The distribution of categorical variables was compared between groups and assessed by the chi squared probability distribution. The probability of developing a poor outcome was evaluated by the Kaplan–Meier method, and the magnitude of association between the treatment group and outcome risk was estimated by the hazard ratio (HR) through Cox proportional hazards-models, adjusting by sex. Statistical analyses and graphics were performed using Stata version 15.0, SPSS Version 21**.**0 (SPSS Inc) and GraphPad Prism 5.0.

## Results

**Figure 1:**
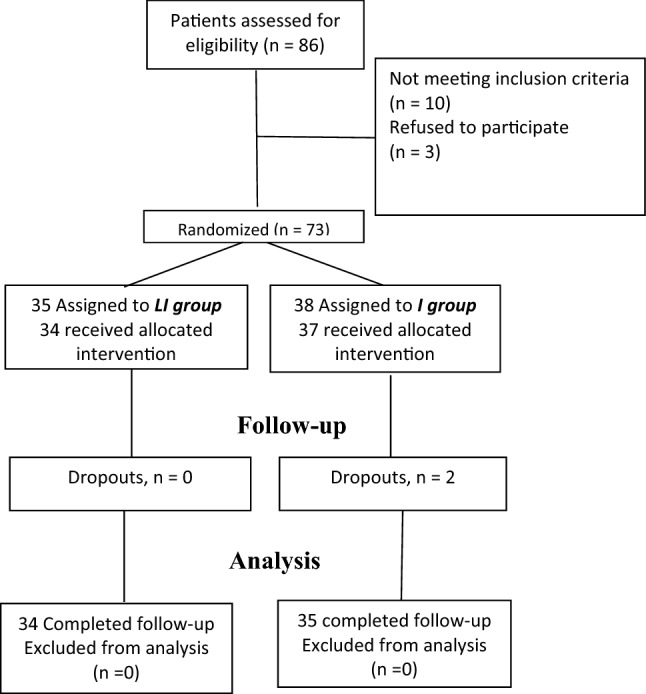
Study profile.

The study was performed between June 2020 and February 2021. Eighty-six hospitalized patients with SARS-CoV-2 infection were screened for the study; 10 patients did not meet the selection criteria and 3 patients refused to participate in the study. A total of 73 patients with SARS-CoV-2 infection plus glucose levels between 140 and 400 mg/dl without assisted mechanical ventilation at hospitalization were randomly assigned to the 5 mg linagliptin daily + basal bolus insulin scheme (*LI group*, n = 35) or the basal bolus insulin scheme only (*I group*, n = 38) for the control of hyperglycemia. Together with the assigned treatment for the control of hyperglycemia, all patients received standard treatment for COVID-19, according to international recommendations. In the *LI group*, only 34 patients received the assigned treatment since one patient required assisted mechanical ventilation before the start of linagliptin administration, and there were no dropouts in this group. In the *I group*, only 37 patients received the assigned intervention since one patient also required assisted mechanical ventilation before the starting of the therapy; 2 patients withdrew from the study in this group because they did not accept glucose monitoring (Fig. 1).

The baseline characteristics of the study groups are presented in Table 1. The age (60 ± 2 vs 57 ± 2 years), number of patients older than 60 years of age (19 vs 19), previous diagnosis of T2D (25 vs 21), HbA1c levels (8.4 ± 0.4% vs 8.9 ± 0.3%), BMI (31.1 ± 1.5 vs 30.8 ± 1.2 kg/m^2^), frequency of comorbidities, initial glycemia (251 ± 21 vs 251 ± 15 mg/dl), disease severity, lipid levels, inflammatory marker levels, and other biochemical variables were similar between the *LI* and *I groups*. There was a greater percentage of males in the *LI group* (26 vs 18, p = 0.030).

**Table 1:** Baseline and clinical characteristics between the study groups.

	I group (n = 35)	LI group (n = 34)	p value
Age (years)	57 ± 2	60 ± 2	0.372
Sex (M/F)	18/17	26/8	0.030
Patients ≥ 60 years of age, n %	19 (54)	19 (56)	0.894
Previous T2D, n (%)	25 (71)	21 (62)	0.395
Duration of diabetes (years)	10 ± 1	11 ± 2	0.475
**Previous comorbidities, n (%)**			
Cardiovascular disease	3 (8)	1 (3)	0.319
Chronic kidney disease	6 (17)	4 (12)	0.387
Stroke	0 (0)	2 (5)	0.238
Smoking, n (%)	3 (9)	7 (20)	0.178
**Glucose-lowering medications, n (%)**			
Insulin	4 (11)	5 (15)	0.900
Metformin	13 (37)	16 (47)	0.373
Sulfonylurea	4 (11)	2 (6)	0.414
HBP, n (%)	18 (51)	21 (61)	0.430
**Antihypertensive drugs, n (%)**			
ACE inhibitors	4 (11)	4 (11)	0.916
ARAII	12 (34)	14 (41)	0.522
b-Blockers	6 (17)	3 (9)	0.302
Diuretics	2 (6)	4 (11)	0.365
Calcium-channels b	7 (20)	6 (18)	0.807
Clinical score (0–7)	4.3 ± 0.10	4.3 ± 0.09	0.873
Weight (kg)	80 ± 3	81 ± 3	0.934
BMI (kg/m^2^)	30.8 ± 1.2	31.1 ± 1.5	0.875
HbA1c (%)	8.9 ± 0.3	8.4 ± 0.4	0.439
Glucose (mg/dl)	251 ± 15	251 ± 21	0.993
Serum creatinine (mg/dl)	1.3 ± 0.2	1.2 ± 0.2	0.505
Total cholesterol (mg/dl)	161 ± 8	165 ± 9	0.722
LDL cholesterol (mg/dl)	86 ± 5	89 ± 8	0.783
HDL cholesterol (mg/dl)	31 ± 2	29 ± 2	0.409
Triglycerides (mg/dl)	216 ± 15	213 ± 16	0.894
Lymphocyte count (mu/l)	903 ± 100	981 ± 93	0.577
CRP (mg/l)	146 ± 21	131 ± 23	0.637
D-dimer (ng/ml)	663 ± 174	1018 ± 266	0.256
LDH (units/l)	478 ± 45	445 ± 37	0.583
Ferritin (ng/ml)	675 ± 98	889 ± 221	0.376
AST (U/l)	59 ± 13	61 ± 12	0.904
ALT (U/l)	49 ± 9	56 ± 14	0.693
Albumin (g/dl)	3.1 ± 0.1	3.0 ± 0.1	0.367
Oxygen saturation (%)	92 ± 0.7	92 ± 0.8	0.838

Data are mean ± SEM.

*CRP* C reactive protein, *LDH* lactate dehydrogenase, *AST* aspartate aminotransferase, *ALT* alanine aminotransferase.

### Mechanical ventilation and mortality

There was a trend toward a shorter duration of hospitalization in the *LI group* (10 ± 1 vs 12 ± 1, p = 0.343) (Table 2). Disease severity, clinical condition and the overall risk of complications were similar between the study groups (Table 2). The use of other support treatments, such as vasopressor amines, prone positioning, antibiotics, dexamethasone, and anticoagulant therapy, was similar between the groups (Table 2). Four patients per group reported previous ivermectin use. During hospitalization, 15 patients required assisted mechanical ventilation, 12 (34.3%) in the *I group* and 3 (8.8%) in the *LI group* (p = 0.010); the patients in the *LI group* had a relative risk for needing assisted mechanical ventilation of 0.258 (95% CI 0.092–0.719, p = 0.010).

**Table 2:** Clinical evolution during hospitalization between the study groups.

	I group (n = 35)	LI group (n = 34)	p value
Days in hospital	12 ± 1	10 ± 1	0.343
Assisted mechanical ventilation n (%)	12 (34.3)	3 (8.8)	0.010
Days from hospital admission to mechanical ventilation	4.5 ± 1.0	5.0 ± 1.5	0.749
Mortality, n (%)	6 (17.1)	2 (5.9)	0.139
Average glucose (mg/dl)	152 ± 3	141 ± 2	0.008
Last fasting glucose (mg/dl)	149 ± 10	122 ± 7	0.033
Last postprandial glucose (mg/dl)	173 ± 12	137 ± 7	0.017
Hypoglycemic events, n	13		11
PaO_2_/FiO_2_ (mmHg/%)	174 ± 12	175 ± 12	0.981
Prone position, n (%)	23 (74)	22 (73)	0.939
Vasopressor amines, n (%)	8 (29)	9 (30)	0.905
Acute renal failure, n (%)	6 (17)	7 (20)	0.678
SOFA score	2.7 ± 0.2	2.5 ± 0.2	0.495
APACHEII score	10.9 ± 1.0	9.8 ± 1.0	0.385
**Main symptoms, n (%)**			
Cought	19 (54)	16 (47)	0.491
Dysnea	26 (74)	21 (62)	0.117
Fever	13 (37)	16 (47)	0.438
Cephalea	11 (31)	10 (29)	0.787
Anosmia	2	3	0.500
**Previous treatment, n (%)**			
Ivermectin	4 (11)	4 (11)	0.960
**Treatment, n (%)**			
Antibiotics	12 (34)	11 (32)	0.590
Dexamethasone	35 (100)	34 (100)	1.000
Anticoagulant	35 (100)	34 (100)	1.000
CRP (mg/L)	72 ± 20	46 ± 15	0.334

Data are mean ± SEM.

*PaO2/FiO2* ratio between partial pressure of arterial oxygen and fraction of inspired oxygen, *SOFA* sequential organ failure assessment score, *CRP* C reactive protein.

**Figure 2:**
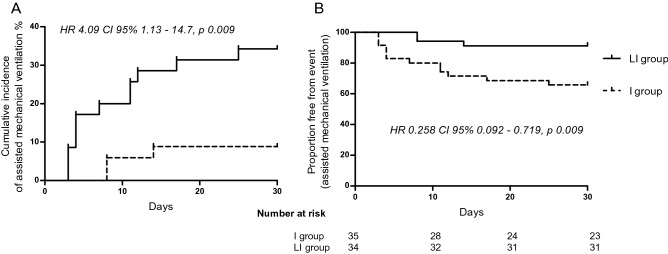
Incidence of assisted mechanical ventilation (**A**), and Kaplan–Meier analysis for assisted mechanical ventilation-free between the study groups (**B**).

Patients in the *I group* had a higher incidence and risk of needing assisted mechanical ventilation than those in the *LI group* (HR 4.09; 95% CI 1.13–14.7; p = 0.030), which persisted after including sex in the regression model (Fig. 2). This suggest that it would be necessary to treat at least 4 patients with linagliptin to avoid the use of assisted mechanical ventilation in one patient. According to intention-to-treat analysis, the lower risk for needing assisted mechanical ventilation in the patients in the *LI group* persisted (RR 0.33; 95% CI 0.12–0.93). On average, the need for mechanical ventilation occurred 4–5 days after hospitalization, with no differences between groups (Table 2). Mortality was 5.9% (n = 2) in the *LI group* and 17.1% (n = 6) in the *I group* (RR 0.34; 95% CI 0.07–1.58; p = 0.196). Assisted mechanical ventilation was significantly associated with mortality since 53% (n = 8) of the patients requiring mechanical ventilation died, while no patient not requiring mechanical ventilation died (p < 0.001). As previously mentioned, there was a greater percentage of males in the *LI group*; however, sex was not associated with the need for assisted mechanical ventilation (8 males and 7 females required mechanical ventilation; p = 0.342) or death (4 males and 4 females died; p = 0.312), however, other factors such as disease severity (SOFA score 3.5 ± 0.3 vs 2.3 ± 0.2; p = 0.002) and pre- and postprandial glucose levels during hospitalization were positively associated with mechanical ventilation, and patients requiring assisted mechanical ventilation had a longer in-hospital stay (18 ± 3 vs 9 ± 1; p < 0.001), a greater increase in basal insulin requirements (at day 4, + 8.3 vs + 2 U/day; p = 0.026) and a greater increase in rapid insulin requirements (at day 2, + 2.8 vs + 0.6 U/day; p = 0.041). Age was not associated with the need for assisted mechanical ventilation since 7 (46%) and 24 (44%) patients who required and did not require mechanical ventilation, respectively, were older than 60 years of age (p = 0.878).

On average, there were no significant differences in oxygen saturation between the study groups, although there was a trend toward lower values in the *I group* (Suppl. Fig. 1A). Respiratory frequency was lower in the *LI group*, and it was significantly different from the basal level at day 6 in the *LI group* only (Suppl. Fig. 1B). The ratio between partial pressure of arterial oxygen and fraction of inspired oxygen (PaO2/FiO2) tended to be lower in the *I group*, especially during the first days, although the difference did not reach statistical significance (Suppl. Fig. 1C). The clinical score was significantly improved (reduced) on day 6 in the LI group, while it was significantly worsened (increased) on day 7 in the I group (Suppl. Fig. 1D). Clinical differentiation was clear between patients who required mechanical ventilation and those who did not, as measured by the PaO2/FiO2 ratio and clinical scores (Suppl. Fig. 1E,F).

### Glucose levels and insulin requirements

**Figure 3:**
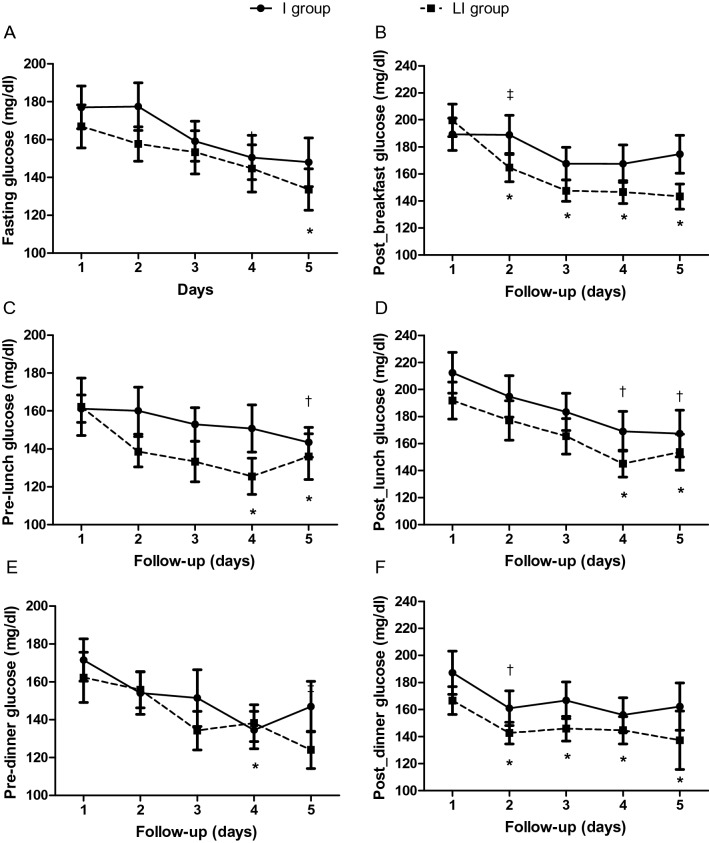
Pre- and postprandial glucose levels during the first 5 days of hospitalization between the study groups. *p < 0.05 vs day 1 in *LI group*; ^†^p < 0.05 vs day 1 in *I group*; ^‡^p < 0.05 for comparison between groups in the change from day 1.

Glucose levels at hospitalization were comparable between the two groups (Table 1). FG levels improved in both groups, but the improvement was significant only in the *LI group* on day 5 (Fig. 3A; p ˂ 0.05); prelunch and predinner glucose levels were also significantly reduced to a higher degree in the *LI group* (Fig. 3C,E).

Postprandial glucose levels during the morning were significantly reduced only in the *LI group*; of note, after 24 h of treatment, the reduction in postprandial glucose levels was significantly higher in the *LI group* than in the *I group* (Fig. 3B, p ˂0.05). Postprandial glucose levels after lunch improved significantly in both groups on days 4 and 5 (Fig. 3D); postprandial glucose levels after dinner were significantly reduced in both groups 24 h after treatment initiation, but this reduction persisted for a significantly longer period of time in the *LI group* (Fig. 3F). Average glucose levels throughout the entire hospitalization were significantly lower in the *LI group* than in the *I group* (141 ± 2 vs 152 ± 3 mg/dl; p = 0.008, Table 2), as were the last fasting (122 ± 7 vs 149 ± 10 mg/dl; p = 0.033) and postprandial glucose (and 137 ± 7 vs 173 ± 12 mg/dl; p = 0.017) levels (Table 2).

**Figure 4:**
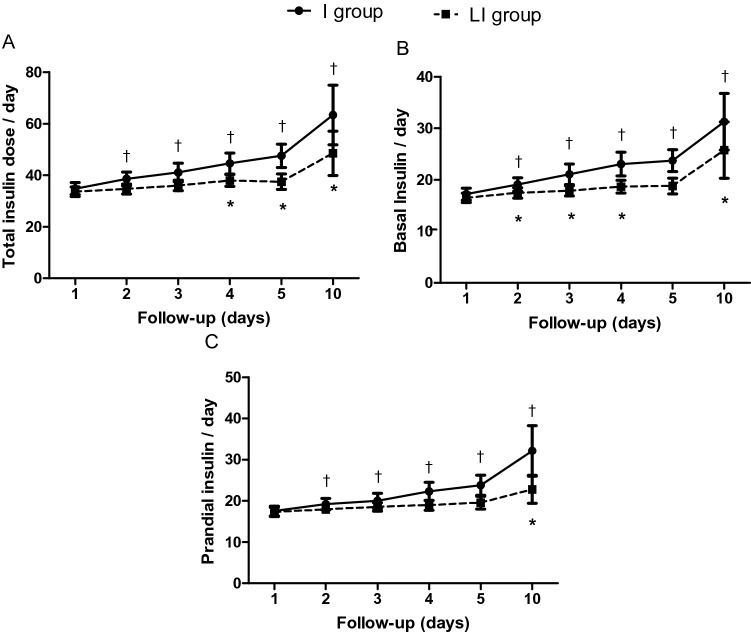
Total (**A**), basal (**B**), and prandial (**C**) insulin requirements between the study groups. *p < 0.05 vs day 1 in *LI group*; ^†^p < 0.05 vs day 1 in *I group*.

On the other hand, insulin requirements, particularly the requirements for prandial insulin, significantly increased with time in both groups, but this increase was higher in the I group than in the LI group (Fig. 4). The frequency and severity of hypoglycemia were similar between groups (11 vs 13 in the I and LI groups, respectively; Table 2).

## Discussion

Severe acute respiratory syndrome coronavirus 2 (SARS-CoV-2) has already affected more than 200 countries. There is no doubt that COVID-19 has placed a considerable stress on most health systems around the world, as there more than 120 million cases and more than 2.7 million deaths as of March 2021. Common metabolic disorders such as T2D and obesity, as well as hypertension, cardiovascular disease, nervous system disease and chronic kidney disease, are known to be risk factors for complications and disease severity in the context of COVID-19^5,47,48^. Obesity and T2D are proinflammatory diseases that often present with other comorbidities, such as hypertension and cardiovascular disease, and systemic endothelial dysfunction. SARS-CoV-2 infection causes an increase in cytokine secretion, which leads to a high risk of vascular hyperpermeability, hypercoagulability, respiratory and multiorgan failure, and death^48^. These pathological abnormalities contribute to insulin resistance and glucose deterioration, which are associated with the use of systemic glucocorticoids and may also cause insulin resistance and hyperglycemia. Particularly in T2D, poor glycemic control at admission has been associated with the worst outcomes in hospitalized patients with COVID-19^5,15^ since hyperglycemia may favor SARS-CoV-2 replication and proinflammatory cytokine secretion, promoting T cell dysfunction and lung epithelial cell death^12^.

The enzyme DPP-4 is a transmembrane glycoprotein that is widely expressed in many tissues and interacts with cellular proteins capable of regulating postprandial glucose via degradation of GLP-1 and stimulating inflammatory and immune responses; DPP-4 cleaves different peptide hormones and bioactive immunomodulators resulting in inactivation of specific biological processes^17,18^. DPP-4 can also be released from the cell membrane and circulate as a soluble amino acid molecule; soluble levels of DPP-4 have been reported to be elevated or normal under different inflammatory conditions^49^. DPP-4 is localized in endothelial and immune cells as well as in type 1 and type 2 pneumocytes^23^, and it has been reported that human DPP-4 may also be a functional coronavirus receptor^31^. Linagliptin is a highly selective and potent DPP-4 inhibitor^50,51^ that increases pancreatic beta cell function and reduces glucagon secretion, thus improving postprandial hyperglycemia^52^.

Previous computational analyses have shown that linagliptin may inhibit SARS-CoV-2 replication^53^, and modeling analysis has shown that the S1 domain of SARS-CoV-2 may bind to human DPP-4^36^. If DPP-4 is a coreceptor for SARS-CoV-2, considering that severity and poor prognosis in COVID-19 are associated with T2D and hyperglycemia, conditions in which DPP-4 is not well regulated, inhibition of DPP-4 could be an alternative therapy for improving metabolic control and prognosis in COVID-19 patients.

Together, these results support a scientific hypothesis linking DPP-4 inhibitors with prognosis in patients with COVID-19. Here, we found a relative lower risk for needing assisted mechanical ventilation of 74% and better improvement in pre- and postprandial glucose levels in hospitalized patients with SARS-CoV-2 and hyperglycemia treated with linagliptin plus an insulin scheme than in those treated only with an insulin scheme; additionally, we found better clinical evolution in pulmonary parameters in patients in the *LI group*. To our knowledge, this is the first randomized clinical trial reporting the effect of a DPP-4 inhibitor in hospitalized patients with SARS-CoV-2 and hyperglycemia. We used assisted mechanical ventilation as a primary outcome, since no patient in the study was with mechanical ventilation at baseline, because it is an early stage of the natural history of the disease, and because mortality is higher in patients once they need assisted mechanical ventilation; in this sense, if the risk of mechanical ventilation is reduced, mortality could be also reduced, and the stress on public health systems would also be lower, since patients with assisted mechanical ventilation requires a more complex health care.

There is little clinical evidence, based on previous observational studies, that the use of the DPP-4 inhibitor, sitagliptin, is associated with reduced mortality in patients with T2D hospitalized for COVID-19^43^. In a retrospective study, the use of sitagliptin at the time of hospitalization was associated with a decreased odds ratio for mortality (OR 0.37; CI 95% 0.23–0.62), and the risk of mechanical ventilation was reduced by 73% in patients who received sitagliptin in comparison with those who only received standard care. In our study, we found a relative risk reduction of 74% for needing mechanical ventilation in patients treated with the combination of linagliptin and insulin, and our findings also showed better improvements in glucose control and pulmonary parameters related to respiratory physiology. We did not find a statistically significant reduction in mortality due to the small sample size, but there was a clinically significant reduction of 66%, which is similar to what was previously reported in a retrospective analysis^43^. Patients in our study were younger than those in previous studies, and we did not find a significant association between age and mechanical ventilation or death, which may have been due to the high prevalence of metabolic abnormalities at a young age in our population and because the study power was limited for this purpose. Besides this, other factors like the use of ACE inhibitors blocking the renin–angiotensin–aldosterone system have been found associated to a reduced mortality in COVID-19 patients^54^, although in our study the proportion of patients with these medications was not different between the study groups.

On average, patients needed mechanical ventilation between days 4 and 5, which highlights the relevance of the first days in the hospital to metabolic control and disease progression, as has also been previously reported^55^. We showed that linagliptin started improving pre- and post-prandial glucose levels in the first 24 h. It seems that in addition to hyperglycemia at admission, glucose level fluctuations and glucose levels during the first week of hospitalization are associated with the worst prognosis in patients with T2D and COVID-19, highlighting the importance of glucose stability and early glucose control in these patients^55–57^. Of note, we found that patients in the *LI group* had a greater reduction in postprandial glucose levels even 24 h after the start of therapy and showed a better improvement in glucose levels at different times of the day. These improvements in glucose levels were seen in patients in the *LI group* besides they required lower basal and prandial insulin doses throughout the entire study. Treatment of hyperglycemia in hospitalized patients mainly consists of insulin administration; however, some reports have associated the previous use of insulin with higher mortality risk in patients with T2D, which could be mostly related to disease severity and other comorbidities^11,58^.

However, although hyperglycemia may have a negative impact on prognosis in hospitalized patients with COVID-19^44^, it seems that the effect on glucose control would not be the only responsible for the beneficial effect of DPP-4 inhibitors in patients with COVID-19, since we saw the improvements in glucose levels during the first 24 h of hospitalization and the need of mechanical ventilation was between 4–5 days. Different mechanisms have been proposed to be associated with the effect of DPP-4 inhibitors in patients with COVID-19. Since it has been reported that soluble DPP-4 levels increase after the administration of different DPP-4 inhibitors to mice^59^, it is possible that DPP-4 inhibitors reduce SARS-CoV-2 virulence by reducing its binding to DPP-4 in the cell membrane and reduce SARS-CoV-2 entry into cells^36^; alternatively, it is possible that a higher abundance of soluble DPP-4 binds SARS-CoV-2, preventing the attachment of the coronavirus to membrane-bound DPP-4 in pneumocytes and other cells, as previously suggested^60^. Computational analysis has shown that linagliptin could be a potential inhibitor of SARS-CoV-2M pro viral cysteine protease^61^. Another proposed mechanism is regulation of the immune response and a reduction in the cytokine storm that occurs following viral entry and that induces the progression of the disease in different tissues via highly selective inhibition of DPP-4 by linagliptin. Experimental studies have reported that linagliptin reduces inflammatory marker levels by inhibiting inflammatory pathways in inflammatory bowel disease^62^. Additionally, linagliptin may improve prognosis by controlling glucose levels improving pancreatic islet function, inhibiting glucagon secretion, and stimulating insulin secretion, achieving better glucose stability in a short time and having a relevant effect on postprandial glucose. Other studies have shown a renoprotective role of linagliptin in animal models by modifying different signaling pathways (collagen type I homeostasis, HNRNPA1, YB-1, thymosin β4 and TGF- β1 and apolipoprotein C1), which could be also involved in the beneficial effect in COVID-19 patients^63,64^.

Mortality has been reported to be higher in males than in females (2.8 vs 1.7%)^65^. Here, we did not find a significant sex-related difference in the number of patients who required mechanical ventilation or the number of patients who died.

Our study has several limitations. The sample size was relatively small, and the study power was not enough to identify differences in mortality, which is the most important outcome in patients with COVID-19; however, a reduction in assisted mechanical ventilation would eventually impact mortality. Due to sample size, the study could be considered as a pilot study and or a proof-of-concept study, which was performed only in two different institutions from the same geographic area. The main strengths of our study are that it was a randomized clinical trial and that we were able to report the detailed progression of clinical and biochemical parameters.

In conclusion, the use of linagliptin in combination with insulin in hospitalized patients with SARS-CoV-2 and hyperglycemia reduces the risk of assisted mechanical ventilation by 74% and improves glucose control and pulmonary parameters related to clinical evolution and prognosis. Further randomized clinical trials with longer durations and larger sample sizes and involving different types of patients are needed to fully elucidate the usefulness of DPP-4 inhibitors in patients with SARS-CoV-2 infection to recommend their use in clinical practice.

## Supplementary Information

Supplementary Figure 1.

## Data Availability

The datasets generated during and analyzed during the current study are not publicly available but are available from the corresponding author on reasonable request.
