# Supplementary material for: Effect of linagliptin plus insulin in comparison to insulin alone on metabolic control and prognosis in hospitalized patients with SARS-CoV-2 infection
Source: Sci Rep. 2022 Jan 11;12:536. doi: 10.1038/s41598-021-04511-1 (PMC8752656; doi:10.1038/s41598-021-04511-1)
Supplement: Supplementary file 1 — Supplementary Figure 1. [file 41598_2021_4511_MOESM1_ESM.pdf]

Effect of Linagliptin Plus Insulin in Comparison to Insulin Alone on Metabolic Control and Prognosis in Hospitalized Patients with SARS-CoV-2 Infection

Rodolfo Guardado-Mendoza MD PhD, Miguel Angel Garcia Magaña MD, Liz Jovanna Martínez Navarro MD, Hilda Elizabeth Macías Cervantes MD, Rodolfo Aguilar-Guerrero MD, Erick L. Suárez-Pérez, Alberto Aguilar-García MD

Short running tittle: linagliptin for SARS-Cov-2 infection

Rodolfo Guardado-Mendoza MD PhD, Research Department Hospital Regional de Alta Especialidad del Bajío, and University of Guanajuato, León, Guanajuato, México, guardamen@gmail.com

Miguel Angel Garcia Magaña MD, Internal Medicine Department, Hospital Regional de Alta Especialidad del Bajío, León, Guanajuato, México, miganggar7@gmail.com

Liz Jovanna Martínez Navarro MD, Internal Medicine Department, Hospital Regional de Alta Especialidad del Bajío, León, Guanajuato, México, falcot23@hotmail.com

Hilda Elizabeth Macías Cervantes MD, Internal Medicine Department, Unidad Médica de Alta Especialidad T1, Instituto Mexicano del Seguro Social, León, Guanajuato, México liz_2787@hotmail.com

Rodolfo Aguilar-Guerrero MD, Internal Medicine Department, Unidad Médica de Alta Especialidad T1, Instituto Mexicano del Seguro Social, León, Guanajuato, México, rodolfo_merhaba@hotmail.com

Erick L. Suárez-Pérez, Department of Biostatistics and Epidemiology, Graduated School of Public Health, University of Puerto Rico, erick.suarez@upr.edu

Alberto Aguilar-García MD, Endocrinology Department Hospital Regional de Alta Especialidad del Bajío, León, Guanajuato, México, betaag@yahoo.com.mx

Corresponding autor: Rodolfo Guardado-Mendoza MD PhD, guardamen@gmail.com , Blvd.Milenio #130, Col. San Carlos la Roncha, León Guanajuato. CP. 37660, Tel.+52 (477) 267 2000.

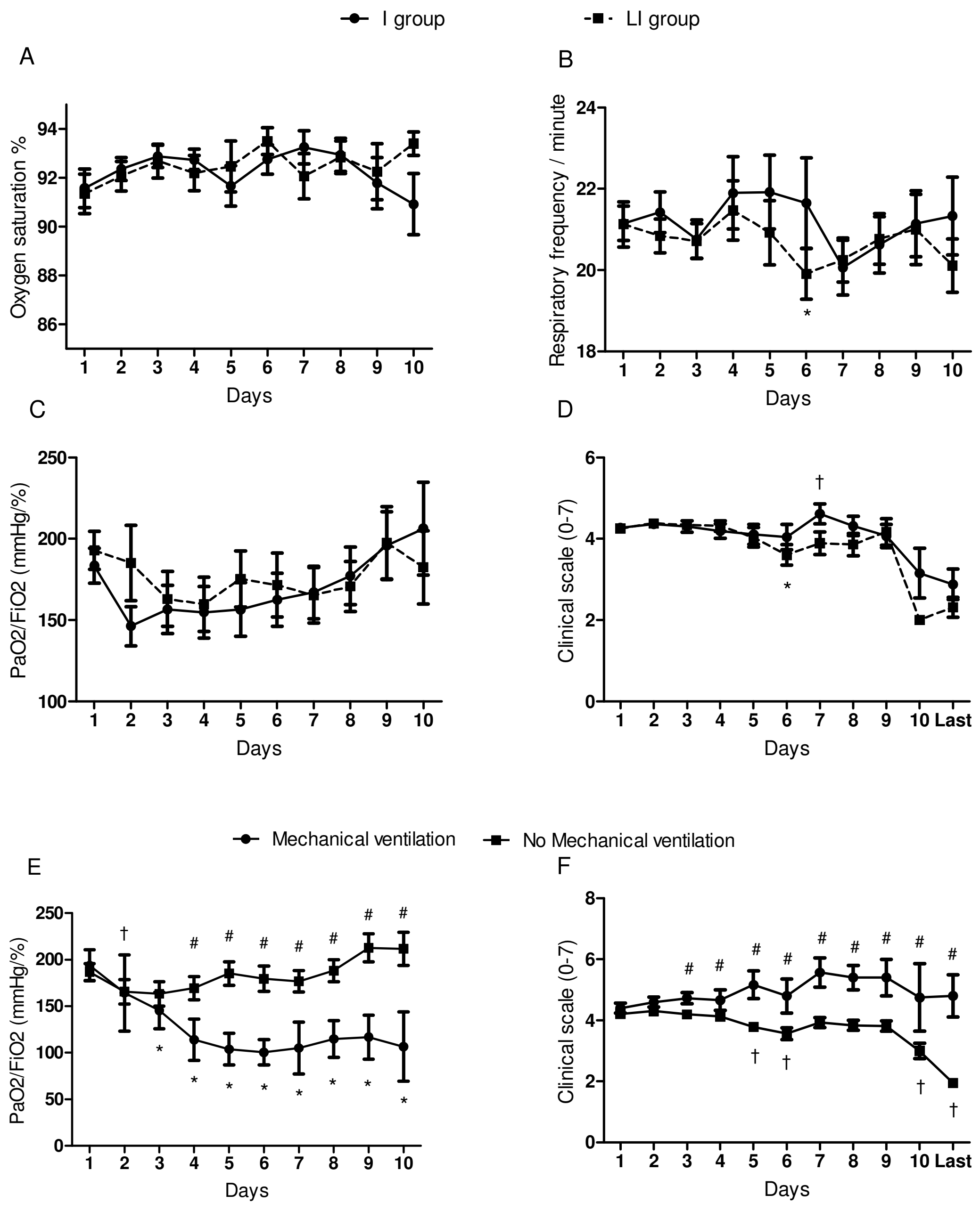

Supplemental Figure 1. Oxygen saturation (A), respiratory frequency (B), PaO2/FiO2 (C), and Clinical scale evolution (D) between the study groups during the first 10 days of hospitalization.

*p<0.05 vs day 1 in *LI group*; †p<0.05 vs day 1 in *I group*. PaO2/FiO2 (E) and Clinical scale evolution (F) between patients *with* or *without mechanical ventilation*. *p<0.05 vs day 1 in patients *with mechanical ventilation*; †p<0.05 vs day 1 in patients *without mechanical ventilation*; # p<0.05 for comparison between patients *with* and *without mechanical ventilation* at different time points.
